# Supplementary figures and images for: Adaptation of a transmitted/founder simian-human immunodeficiency virus for enhanced replication in rhesus macaques
Source: PLoS Pathog. 2023 Jul 3;19(7):e1011059. doi: 10.1371/journal.ppat.1011059 (PMC10348547; doi:10.1371/journal.ppat.1011059)

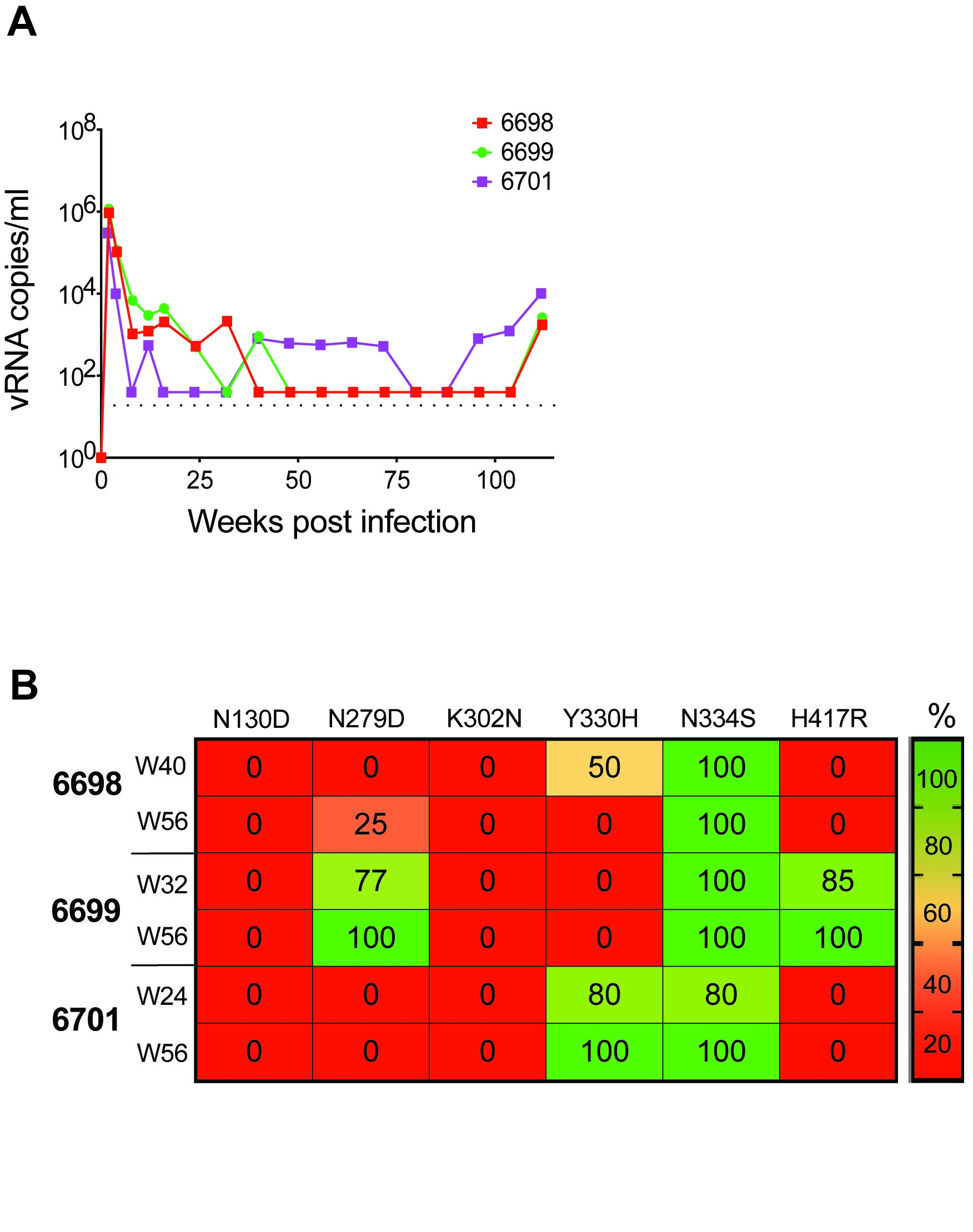

Supplement: S1 Fig — (A) Viral kinetics in three RMs infected with SHIV.C.CH505 with lower viral loads. The dotted line indicates the assay’s limit of detection (LoD) (62 copies/ml). (B) Frequency of signature mutations determined by SGS. (TIF) [file ppat.1011059.s001.tif]

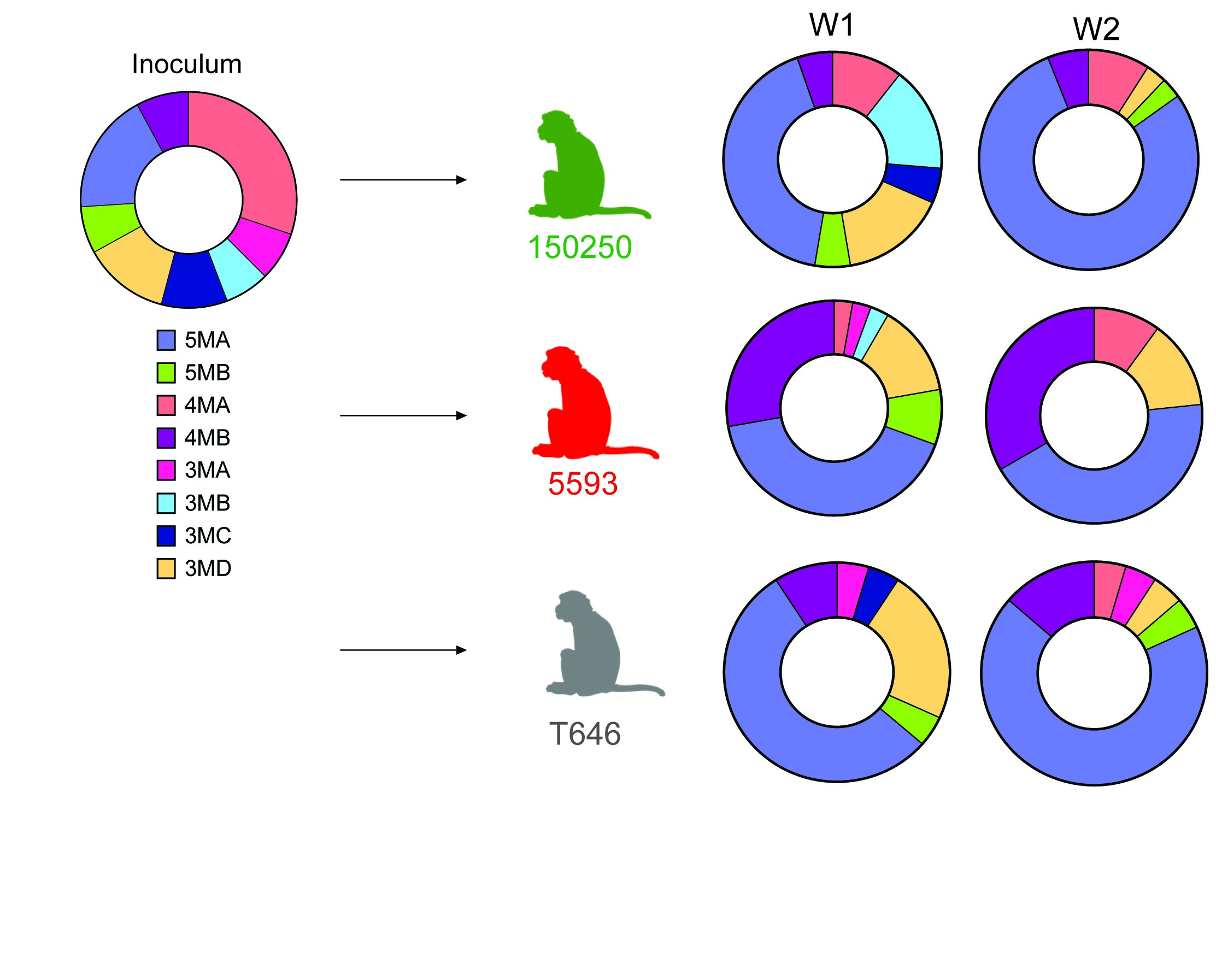

Supplement: S2 Fig — All eight variants were identified, with the 5MA variant replicated most efficiently as observed by Illumina deep sequencing (Fig 7). No recombinant viruses were identified. The distribution of variants in the inoculum (left side) was determined by deep sequencing and reproduced from Fig 7. S2 Fig was created with BioRender.com. (TIF) [file ppat.1011059.s002.tif]

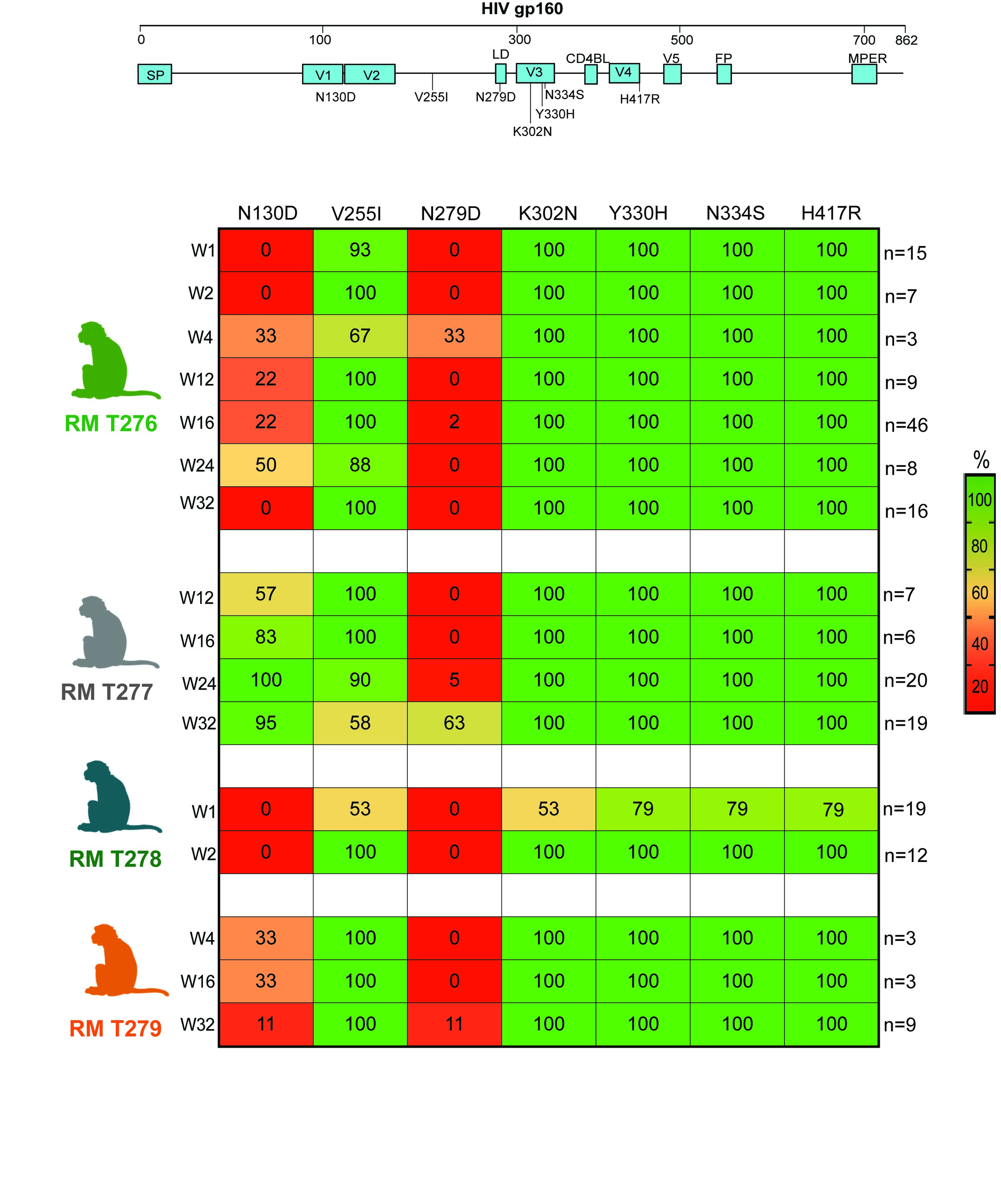

Supplement: S3 Fig — The frequency of each of the 5 signature mutations, as well as N130D and N279D, is shown in the heatmap, with the number of SGS sequences shown at right. S3 Fig was created with BioRender.com. (TIF) [file ppat.1011059.s003.tif]

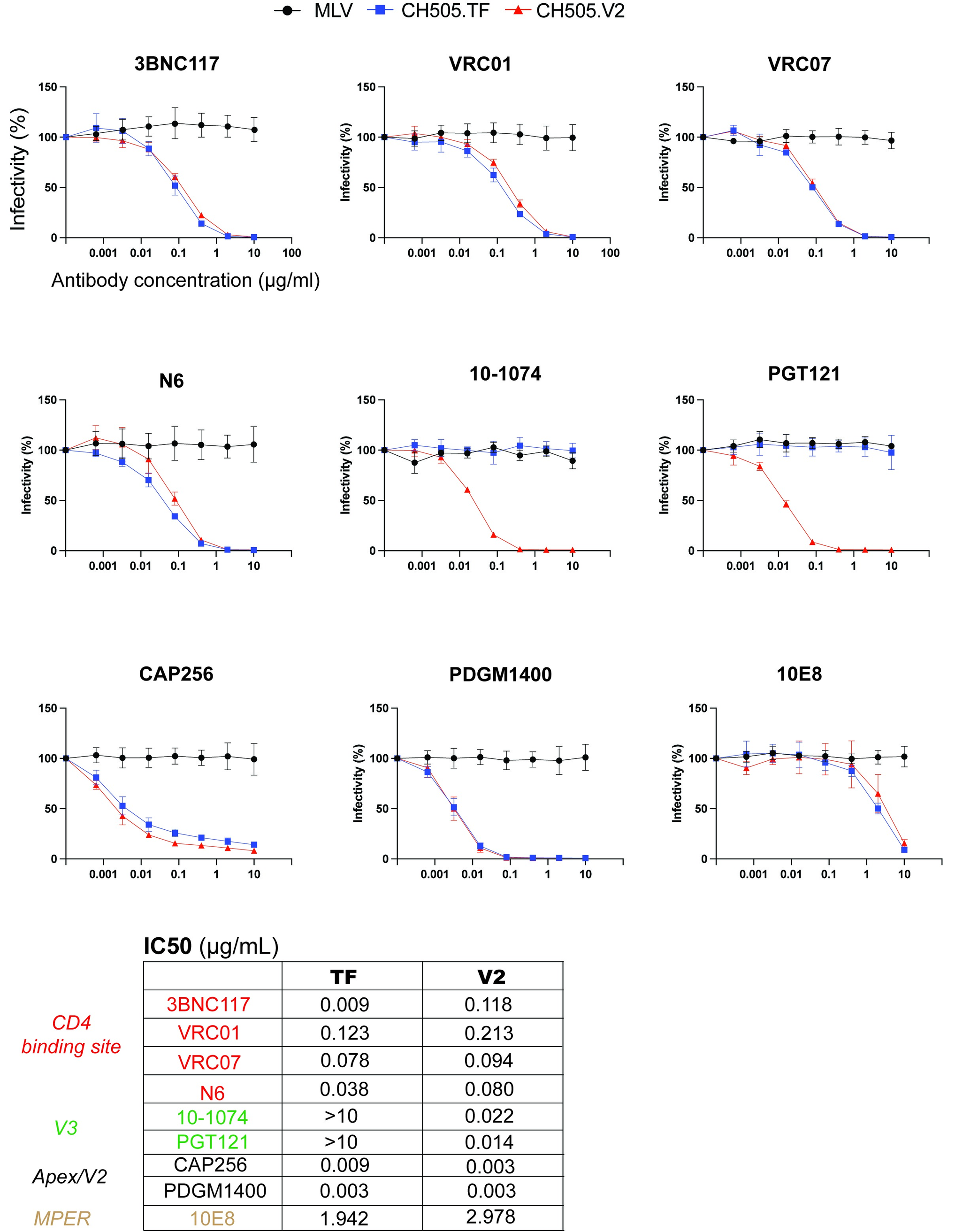

Supplement: S4 Fig — The concentration (μg/mL) of bnAb conferring a 50% reduction in infectivity (IC50) is shown in the table for each bnAb. (TIF) [file ppat.1011059.s004.tif]
