# Supplementary material for: Adaptation of a transmitted/founder simian-human immunodeficiency virus for enhanced replication in rhesus macaques
Source: PLoS Pathog. 2023 Jul 3;19(7):e1011059. doi: 10.1371/journal.ppat.1011059 (PMC10348547; doi:10.1371/journal.ppat.1011059)
Supplement: Attachment — Submitted filename: Comments_to_Reviewers.docx [file ppat.1011059.s007.docx]

Detailed responses to reviewers’ comments.

**Part I - Summary**

Reviewer #1: In this very well-written manuscript, Bauer and colleagues thoroughly outline the rationale for and the development and in vitro / in vivo validation of a novel T/F SHIV for use in nonhuman primate cure studies with interventions that target the HIV envelope. Using plasma from macaques infected with SHIV.C.CH505 the authors apply single genome sequencing to find mutations associated with higher set point viral loads (as low viral set points and spontaneous control of viremia has been a criticism of this and other SHIV strains). The impact of these mutations in terms of replication and antibody sensitivity is then tested individually and in combination using infectious molecular clones. The authors identify 5 mutations within or near the CD4 and coreceptor binding sites that distinguish the original SHIV.C.CH505 from an adapted SHIV.C.CH505.v2 and demonstrate that this mutated virus displays enhanced virus entry and replication. Importantly, spontaneous control of viral replication (in absence of ART) was not found. After 6 months of ART, SHIV.C.CH505.v2 rebounded soon after ART interruption and set point viral loads after rebound were similar to or ~1 log lower than pre-ART set point.

Reviewer #2: In this report, Bauer et al., have illustrated the development of a minimally adapted simian-human immunodeficiency virus, named SHIV.C.CH505.v2. They have conducted detailed sequence analyses to first identify a signature of envelope mutations associated with higher viremia in infected rhesus macaques. And used short-term in vivo mutation selection and competition to identify an isolate with five amino acid changes that improved in vivo virus replication fitness in macaques. They show that this minimally adapted SHIV had improved virus entry, enhanced replication, and preserved neutralization profiles in vitro. Also, their results demonstrate the virus rapidly outcompeted the parental SHIV and most importantly, persisted during suppressive antiretroviral therapy by showing rebound at treatment interruption.

Overall, the manuscript is very well written, clearly organized and the results are important in the development of an enhanced tool for NHP studies of HIV-1 transmission, pathogenesis, and cure. My minor comments are listed below:

1. Lines 84-86: “However, differences between SIVmac and HIV-1, including highly divergent envelope (env) sequences, distinct accessory genes, and variable set-point viremia, immunopathogenesis and natural history, challenge the direct translation of NHP study findings to people living with HIV-1 (PLWH).” This statement conveys that the immunopathogenesis of SIVmac and HIV-1 are distinct, which can be misleading to the general reader as the SIVmac model is a widely used and well-accepted model of HIV immunopathogenesis. I would suggest removing “immunopathogenesis” from the multiple other factors listed here as being divergent between SIVmac and HIV-1.

Response: We appreciate this comment and have amended these statements to remove the term “immunopathogenesis” to avoid confusion (see introduction).

2. Line 180: Figure.2 legend mentions that a setpoint of > 10^4 copies/ml was used as the criterion for defining high VL, but the VL fluctuate a lot and some high VL seem to be below that threshold for many time-points. Was a particular time-point used? Please clarify.

Response: We have more clearly defined that virus setpoint was an “average” over VLs collected after week 12 (lines 186-189).

3. Lines 318-319: “All animals became productively infected with peak VL ranging from
10^4 to 10^6 c/ml (Fig. 7B), though it is probable that the peak VL was not captured with this sampling strategy”. What time-point was this? If it is earlier than 2 weeks, then the authors may just state the time-point and add that the peak viremia was likely higher than this.

Response: We agree this was unclear. We have removed this comment as it is conjecture.

Reviewer #3: There is great interest in the use of bNAbs for HIV therapeutics and cure, however, many of the available SHIVs do not have replication kinetics similar to that of HIV. Bauer et al includes data from previous studies to identify a panel of mutations in env that were repeatedly observed in different animals and suspected to improve replication. They then go on to evaluate these mutations in vitro to assess their replication and neutralization properties. They then evaluated combinations of these mutations both in vitro and in vivo. Finally, in order to assess whether the new model is an improvement, they competed the new and parental model in vivo in four animals. Unfortunately, the in vivo replication of these clones was only evaluated over the first four weeks of infection. As a result, it is impossible to assess whether the new model is an improvement over extended periods of untreated infection. The authors have identified an intriguing new model that warrants additional investigation and may prove to be useful for future studies.

Response: As discussed below, we better describe the limitations of the in vivo competition approaches, with additional discussion of limited numbers in the results section and in the limitations section of the discussion.

**Part II – Major Issues: Key Experiments Required for Acceptance**
Please use this section to detail the key new experiments or modifications of existing experiments that should be absolutely required to validate study conclusions.

Generally, there should be no more than 3 such required experiments or major modifications for a "Major Revision" recommendation. If more than 3 experiments are necessary to validate the study conclusions, then you are encouraged to recommend "Reject".

Reviewer #1: The major criticism of this work is the small number of macaques used for the final experiment of SHIV.C.CH505.v2 persistence on ART and rebound after ART interruption. They start with 4 macaques total, but only 2 were treated with ART for a meaningful period of time and then allowed to rebound. This limitation must be acknowledged. Additionally, suppression of viremia within 2 weeks of ART initiation is unusual for both SIV and HIV infections, even with integrase-containing regimens. This finding should be considered in the Discussion of this model.

Response: We more fully acknowledge this limitation in the results and discuss it directly in a limitations section of the discussion (lines 566-571). We also discuss the rapid suppression of viremia on ART and other relevant discrepancies between this SHIV model and HIV-1.

Reviewer #2: None.

Reviewer #3: • The authors work very hard to characterize the signature mutations that they identified, but then spend much less time comparing SHIV.C.CH505.V2 to SHIV.C.CH505 TF. There is extensive information about the infection dynamics of SHIV.C.CH505 TF. As a result, one of the most important ways to evaluate the new model is to compete it against SHIV.C.CH505 TF. The authors do this in four animals, but only assess the relative frequency of each clone up to week 4. At this time the population is dominated by SHIV.C.CH505.V2 but after the host immune response arises the relative fitness of the clones could change. In addition, the high viral loads in figure 8B cannot just be attributed to SHIV.C.CH505.V2 alone. The manuscript would be much stronger with thorough analysis of the viral population after a longer period of time. Given that recombination and evolution are likely to have fundamentally changed the population, this would require SGS sequencing and careful analysis of the population. In the absence of this data the following statement doesn't seem accurate/precise "In vivo, the minimally adapted virus rapidly outcompetes the parental SHIV". A more accurate description would be that "In vivo, the minimally adapted virus outcompeted the parental SHIV over the first few weeks of infection, but their relative fitness was not evaluated over extended periods of untreated infection". Similarly, it would be very helpful to know the relative frequencies of the different combinations of clones (Figure 7) after a longer period of time.

Response: To continue the assess the relative fitness of the two variants in the *in vivo* competition shown in Figure 8, we have performed additional single genome sequencing of gp160 on longitudinal time points. As suggested by the reviewer, we opted for SGS over deep sequencing approaches to better control for recombination within the diversifying virus populations. Unfortunately, our choices of available plasma samples were limited in some RM due to storage issues, but we did have later time points for 3 of the 4 RM. Our results are discussed in lines 408-417 and summarized in the new supplemental figure **Fig.S3**. In short, we generated a total of 212 SGS *env* sequences, which demonstrate the overall persistence of the 5 signature mutations through 32 weeks. We did observe a small frequency of reversion of the mutation at 255 (I🡪V), generally paired with the de novo N279D mutation. These longitudinal sequencing results support our assertion that V2 outcompetes the TF. In addition, linkage of the V2 mutations (V255I and N279D) supports the proposed similar mechanism of compensating for changes incurred with the V3 mutations (see discussion).

• I think that multiple names are being used for the same stocks (SHIV.C.CH505, SHIV.C.CH505.TF, TF SHIV.C.CH505, TF SHIV, CH505-TF, parental SHIV.C.CH50, TF). If these are the same, then it would be easier if a single name was used. If they aren't the same then the text needs to be clarified.

Response: We appreciate this comment. We have attempted to clarify the naming scheme by using “SHIV.C.CH505” when referring to historical studies and “SHIV.C.CH505.TF” for future experiments and when directly comparing to SHIV.C.CH505.v2 or other mutants.

• Was a UMI used in the MiSeq analyses? If not, how can the frequency of an allele be assessed without knowing the number of RNA templates sequenced? This is particularly problematic when populations contain many clonal sequences making it impossible to determine whether the population contains many identical genomes or the same small number of genomes are being sequenced many times. In the absence of a UMI, the frequency of genomes cannot be accurately assessed.

Response: We agree with the reviewer that NGS sequencing methods employing UMI would have been more robust. While acknowledging the value of UMI to characterize the denominator of sequenced templates fully, we feel our results remain valid for several reasons:

1. We did estimate the number of cDNA templates in each sequenced sample, aiming to have a known ratio of input template and a standardized, minimum target of sampling depth.
2. We performed multiple replicates of the NGS for each scientific question. For example, to model the kinetics of growth between V2 and TF variants, we performed multiple sequencing runs on the samples from multiple time points for each of 4 RM. From these, sequencing results were aligned in frequencies, supporting findings from the in vitro competition and single genome sequencing of the same plasma samples. We did not have any unexpected or outlying results—across replicates, or related samples—compared to SGS or replication results. Similarly, in Figure 7, we sampled 3 RM over 3 timepoints with technical replicates of each sample. We would have had to have significant jackpotting across all samples to conclude other than finding 5MA to be the fittest variant.

Thus, we acknowledge the validity of the reviewer’s comment. But, given the number of replicates with similar findings, the lack of unexpected results, and the support of multiple other lines of evidence, we feel these NGS approaches are reasonable.

• It was challenging to identify whether the data were new or previously published. Specifically, was the data in figures 1, 2 and 3 previously published? If so, it may streamline the study to say that the 7 signature mutations repeatedly emerged during previous infection experiments.

Response: Some of the sequences in fig 1 and 3 were previously published, but not in this format. Fig 2 sequences have not been published. We have better clarified this in the methods. We understand the reviewers point, but feel the inclusion of these sequences is essential to the story.

• The source of the clones should be clarified. When describing Figure 5 the authors state " We next generated IMCs encoding combinations of the six signature mutations in a newer TF SHIV backbone, 3C, which contains deletions in the tat and gp41 env reading frames that were selected in vivo in multiple RM infected with distinct TF SHIVs and conferred a fitness advantage in vitro compared to the previous backbone (63), and has been incorporated into current iterations of TF SHIVs (54)." That suggests that all of the signature mutations were identified in experiments infecting RM with the original SHIV.C.CH505 IMC and its descendants that were subsequently passaged in RM. While the mutations accumulated in the older backbone I think that they were often (always?) characterized using the newer 3C backbone. This should all be discussed more clearly. In addition, it seems possible that the mutations may behave differently in the two backbones. It is definitely worth discussing the implications of this change and specifying the source of the variants used in the different experiments.

Response: We have clarified which clones contain which backbone and better referenced the distinction between backbones. We have also attempted to better clarify that for any in vitro or in vivo comparison, the competing clones contain the same backbone. The 3C backbone was determined by examining sequence evolution in multiple TF SHIVs bearing distinct TF Env cassettes—these mutations are apart from the gp120 region that is the focus of this paper. For this paper, all experiments, except the in vitro comparison of SHIV.C.CH505.TF vs. single mutants (described in Fig 5B and C) were completed with the 3C backbone. Notably, the fold-change comparisons in Fig 5C and 5E compared SHIVs with the same backbone (original in 5C, 3C in 5E).

**Part III – Minor Issues: Editorial and Data Presentation Modifications**
Please use this section for editorial suggestions as well as relatively minor modifications of existing data that would enhance clarity.

Reviewer #1: 1. Page 6, line 152 appears to be missing a reference in which SHIV.C.CH505 was used in adult macaques (Dashti et al J Virol 2020).

Response: Thank you for noticing this. This reference has been added and the number of total adult animals and frequency of control was updated accordingly.

2. Page 6, line 152: correct SHIC to SHIV.

Response: addressed.

3. Page 7: text referring to Fig 2B defines fully penetrant as 100%; however, the 3 mutations that are stated to meet this criterion (K302N, Y330H, H417R) are shown at a frequency of 80%. The text should be revised to avoid confusion.

Response: In Figure 2, the table lists the number of RM in which all sequences contain the specified mutation. Thus, 80 refers to 80% of RM having sequencing showing complete penetrance (100% of sequences) with this mutation. We have attempted to make this more clear in the text and Figure legend.

4. Page 7, line 181: correct 80% to 83%.

Response: Addressed.

5. Fig 1C legend (and other legends describing similar figures): specify that percentages are calculated from the total number of sequences generated (to distinguish from the percentage of animals with the specific mutation detected).

Response: This has been done. For Figure 2, we report the percentage of animals with full penetrance of mutation. For the others, we report the frequency of mutations within generated sequences.

6. Highlighter plots: what do the black ticks represent?

Response: We better clarify the highlighter plot, including what the master virus is and that black tics are deletions.

7. Fig 3: the rationale for performing SGS out to week 48 in RM 5695 and week 24 in RM 5181 (and not from later time points) should be clearly stated.

Response: Analyses focus on the first six months of infection in both RM. To clarify, we have removed the display of the w48 sequences from the RM5695.

8. Fig 3B: there appear to be additional mutations that arise beyond the 7 shown in Fig 3C. Page 8, Lines 202-203 states that no other mutations rose to full penetrance by week 24 although the data are not presented to evaluate this statement. Perhaps a table could be included with all of the mutations and their penetrance at week 24?

Response: In RM5695, other mutations arise by week 24. However, these mutations are not fully penetrant. The highlighter plot (with the solid red lines) indicates the fully penetrant mutations. Additionally, in response to the reviewer’s request, we have added a supplemental file with a complete list of mutations observed by week 24 in animal RM5695 (supplemental file).

9. Results text describing Fig 6 should detail the panels being described (A-F) to enhance clarity.

Response: We have done this.

10. Fig 6C-D: CH505.v2 is included here but has not yet been described in the text. Suggest removal.

Response: We have done this.

11. Page 13, line 371: specify if the individual or combination variants are used for infection.

Response: We used the combination of mutant variants and have clarified this in the text (page 13).

12. Page 13, line 319: explain why the peak viral load may not have been captured with the sampling strategy (that occurred “frequently over the first 4 weeks of infection”) or remove this phrase.

Response: We have removed this phrase as it was conjecture.

13. Fig 7A and 8A, shouldn’t the doughnut shapes representing the inoculum show an equal proportion of each variant?

Response: The inoculum was made with equal portions of each variant, as measured by p27 Ag, then sequenced by NGS to characterize the proportion. While both methods are subject to error, we chose to display the relative proportion of each variant as reflected by NGS sequencing for consistency, because sequencing is the method used to characterize proportions in the later time points..

14. Was there any difference in peak viremia for v2 with and without barcoding? The x-axis scale of Fig 8B make this difficult to assess.

Response: We have observed no differences in viral kinetics of barcoded vs non-barcoded viral kinetics at peak or any other timepoint in our experiments. Similarly, identical kinetics have been seen in the published literature of other barcoded SIV/SHIVs (Fennessey PLoS Pathogs 2017; ref 66).

15. Results text describing figure 9A should specify the exact number of weeks until rebound (and the definition of rebound used) after ART interruption.

Response: Viral kinetics of post-ATI rebound are now better described in the text (lines 466-467).

16. Limit of detection of plasma viral load assay used should be added to the Methods and shown on the figures.

Response: the LoD was added as a dotted line in all the figures and described in methods and legends.

17. I did not see legends for the supplementary figures but may have missed them.

Response: The legends to the supplementary pictures were after the references in the original submission file.

Reviewer #2: 1. Lines 84-86: “However, differences between SIVmac and HIV-1, including highly divergent envelope (env) sequences, distinct accessory genes, and variable set-point viremia, immunopathogenesis and natural history, challenge the direct translation of NHP study findings to people living with HIV-1 (PLWH).” This statement conveys that the immunopathogenesis of SIVmac and HIV-1 are distinct, which can be misleading to the general reader as the SIVmac model is a widely used and well-accepted model of HIV immunopathogenesis. I would suggest removing “immunopathogenesis” from the multiple other factors listed here as being divergent between SIVmac and HIV-1.

Response: we have removed the term “immunopathogenesis,” as suggested.

2. Line 180: Figure.2 legend mentions that a setpoint of > 10^4 copies/ml was used as the criterion for defining high VL, but the VL fluctuate a lot and some high VL seem to be below that threshold for many time-points. Was a particular time-point used? Please clarify.

Response: we have better defined that average VL in timepoints after study week 12 was used as the criterion.

3. Lines 318-319: “All animals became productively infected with peak VL ranging from
10^4 to 10^6 c/ml (Fig. 7B), though it is probable that the peak VL was not captured with this sampling strategy”. What time-point was this? If it is earlier than 2 weeks, then the authors may just state the time-point and add that the peak viremia was likely higher than this.

Response: We have removed this statement.

Reviewer #3: • There are two sequencing approaches described in this manuscript (SGS and MiSeq), but it's not always clear which is being used. I think that the frequency data in Figs 1C, 2B, 3C is based on SGS and 7A, 8A and 8C are all MiSeq. I would specify this in the legends. Also, I would list the limit of detection for the different assays.

Response: The method of sequencing is now defined in each figure legend. The limit of detection of the virus quantification assay was added to the viral load figures.

• The authors are describing a new SHIV with many properties that may make it desirable for future studies. The study culminates with an experiment infecting four RM with the newly developed SHIV.C.CH505.v2. At the very end of the results they mention that 2 of the 4 animals had to be euthanized due to wasting after approximately 5 years of therapy. In addition, RM 5695 and RM 5181 had to be euthanized between 1 and 2 years after infection. I would add symbols to the viral load graphs indicating when the animals were euthanized. This could be helpful information as researchers are evaluating whether this new model is appropriate for the study that they are planning.

Response: We have added symbols to the VL figures to help identify time of necropsy.

• Please list the specific env that is serving as the master sequence in the highlighter plots in Fig 1A and 3B. SHIV.C.CH505 seems the best choice to me.

Response: SHIV.C.CH505.TF is the master sequence, as now described in the figure legend.

• The authors should provide justification for the various assays. Why were some done with RM CD4+ T cells, others with ZB5 cells and others with TZMs?

Response: We used RM CD4 T cells for in vitro replication of various SHIVs, TZM.bl for standardized neutralization assays, and a ZB5 cell line that expresses RM CD4 and CCR5 to discern entry into RM cells. We have provided a better explanation of this in the text.

• It would be interesting to discuss why the entry patterns in Fig 5C and 6B are so different.

Response: This is an interesting question. In theory, the difference between the two is either due to i) post-entry effects of the mutations (as 5C depicts the entire multi-step replication process of the single mutant viruses in RM CD4 T cells, while 6B reflects just entry into ZB5 cells expressing RM CD4 and CCR5), or ii) distinctions between two imperfect assay systems. In practice, results from these two assessments show similar trends across mutants, with the exception of the virus containing K302N in isolation. These results are highlighted in the discussion of the impact of K302N and V255I (paragraphs starting on line 514)

• The authors state that the signature mutations "likely arose via escape from early immune responses or reversion to group M consensus". What evidence suggests that 255, 279 and 417 arose this way?

Response: The reviewer makes a good point, as we do not have any evidence that the mutation at 255 is related to either adaptive immune escape or group M reversion. For the other mutations, there is literature to support this description. The mutation at 417 described as CTL escape in CH505 and as group M reversion. (Liao H-X, et al. Co-evolution of a broadly neutralizing HIV-1 antibody and founder virus. Nature. 2013;496(7446):469-76; Roark RS et al., Science. 2021;371(6525):eabd2638.)

N279D is associated with nAb escape in CH505 and with CD4bs bnAb-escape (Lynch RM et al, JVI. 2015;89(8):4201-13.). We have modified our statement accordingly.

• The discussion of the signature mutations (lines 225-235) is confusing. The authors state "Three signature mutations (K302N, Y330H, N334S) encode amino acids that are highly conserved (67-99%) (60)" but go on to state "but the signature mutation residues are less commonly seen than the TF SHIV.C.CH505 amino acid (255 and 279), or neither mutation is common (417)."

Response: In this section, we attempted to describe the first 3 mutations: K302N, Y330H, N334S, that are reverting to Group M consensus. The following 3 mutations: V255I, N279D, H417R, are NOT reverting to Group M consensus. We have revised this section to clarify which mutations are being discussed.

• Is sensitivity to two bNAbs sufficient to assess the neutralization tier? My understanding was that the standard method also involved assessing sensitivity to polyclonal IG (PMID: 19939925)

Response: The reviewer raises a good point, as the classic definition of virus “tier” relates to its sensitivity to plasma from PLWH. Here, we are testing a surrogate—sensitivity to mAbs targeting epitopes (linear V3 and CD4-induced epitopes) that should be masked within a functional virus replicating in vivo. We believe this is a relevant surrogate as it demonstrates that some mutations do “open” the Env to expose these epitopes, while others do not. We have amended the text to reflect testing a surrogate for retained Env conformation and tier status (lines 340-342).
