# Supplementary material for: Adaptation of a transmitted/founder simian-human immunodeficiency virus for enhanced replication in rhesus macaques
Source: PLoS Pathog. 2023 Jul 3;19(7):e1011059. doi: 10.1371/journal.ppat.1011059 (PMC10348547; doi:10.1371/journal.ppat.1011059)
Supplement: Attachment — Submitted filename: June_20_2023_Responses_to_Reviewers_Comments.docx [file ppat.1011059.s008.docx]

Detailed responses to reviewers’ comments.

Reviewer #3: 1. I understand the author's justification for presenting frequency data from deep sequencing analyses that lack UMIs. It would be helpful to describe those justifications in the limitations section in the discussions. This would help the average reader understand the rationale behind their experimental design.

We have addressed this by adding a new paragraph in the limitation section of the revised manuscript.

2. Panels in Fig 5 are discussed out of order

We have revised the legend in Fig.5 to better clarify the figures and panel description.

3. The following sentence should be clarified "Thus, we used the SGS to screen for evidence of in vivo recombination. SGS env sequences from weeks 1 and 2 (n=162) identified all eight inoculum variants, but no in vivo recombinants (Fig S2); thus, we excluded recombinant sequences from the Illumina sequencing analyses."

We have reformulated this sentence for better clarity:

Thus, we used the SGS to screen for evidence of in vivo recombination. SGS env sequences from weeks 1 and 2 (n=162) identified all eight inoculum variants with no evidence of in vivo recombinants (Fig S2); thus, we excluded the possibility of high frequency in vivo recombination, and excluded in vitro recombinant NGS sequences from analyses.
